# Mapping of the US Domestic Influenza Virologic Surveillance Landscape

**DOI:** 10.3201/eid2407.180028

**Published:** 2018-07

**Authors:** Barbara Jester, Joy Schwerzmann, Desiree Mustaquim, Tricia Aden, Lynnette Brammer, Rosemary Humes, Pete Shult, Shahram Shahangian, Larisa Gubareva, Xiyan Xu, Joseph Miller, Daniel Jernigan

**Affiliations:** Battelle–Atlanta, Atlanta, Georgia, USA (B. Jester, J. Schwerzmann);; Centers for Disease Control and Prevention, Atlanta (D. Mustaquim, T. Aden, L. Brammer, S. Shahangian, L. Gubareva, X. Xu, J. Miller, D. Jernigan);; Biomedical and Advanced Research and Development Authority, Washington, DC, USA (R. Humes);; University of Wisconsin, Madison, Wisconsin, USA (P. Shult)

**Keywords:** diagnosis, fluorescent antibody technique, immunoassay, influenza, nucleic acid amplification techniques, genetic sequence analysis, pandemic planning, surveillance, vaccine virus selection, United States, viruses

## Abstract

Influenza virologic surveillance is critical each season for tracking influenza circulation, following trends in antiviral drug resistance, detecting novel influenza infections in humans, and selecting viruses for use in annual seasonal vaccine production. We developed a framework and process map for characterizing the landscape of US influenza virologic surveillance into 5 tiers of influenza testing: outpatient settings (tier 1), inpatient settings and commercial laboratories (tier 2), state public health laboratories (tier 3), National Influenza Reference Center laboratories (tier 4), and Centers for Disease Control and Prevention laboratories (tier 5). During the 2015–16 season, the numbers of influenza tests directly contributing to virologic surveillance were 804,000 in tiers 1 and 2; 78,000 in tier 3; 2,800 in tier 4; and 3,400 in tier 5. With the release of the 2017 US Pandemic Influenza Plan, the proposed framework will support public health officials in modeling, surveillance, and pandemic planning and response.

Influenza viruses cause a substantial burden of illness each year in the United States, estimated at 9.2–35.6 million cases of infection, 4.3–16.7 million clinic visits, 140,000–710,000 hospitalizations, and 12,000–56,000 deaths (*1*). To monitor these constantly changing viruses, the Centers for Disease Control and Prevention (CDC), in collaboration with public health partners, collects and analyzes data from multiple surveillance systems (*2*). These efforts track currently circulating influenza viruses, identify novel influenza viruses of public health importance, monitor antiviral drug susceptibility, and characterize circulating seasonal viruses for guiding influenza vaccine virus selection.

The data and specimens used for influenza virologic surveillance originate from ambulatory patient care facilities, academic and community hospital laboratories, public health laboratories, and commercial laboratories. Recently, CDC has initiated efforts to improve the efficiency of national virologic surveillance and to introduce next-generation, whole-genome sequencing into routine activities. As a first step in this process, we explored existing influenza testing practices and constructed a comprehensive overview of the US virologic surveillance landscape. We evaluated key elements of the system and present a framework for analyzing system strengths and limitations. These findings can be used for informing future modeling efforts, ongoing rightsizing of surveillance, and preparing for a surge in testing during a pandemic response (*3*). In addition, the recently revised 2017 Pandemic Influenza Plan from the US Department of Health and Human Services has initiated a cascade of pandemic influenza plan revisions among other federal, state, and local government partners (*4*). Our proposed framework will support these efforts, providing a common diagnostic operating picture for all levels of influenza testing.

## Methods

To characterize the specimen and data flow used to inform influenza virologic surveillance, we conducted open-ended interviews with clinicians, state public health laboratory (PHL) directors, epidemiologists, and laboratorians from CDC and the Association of Public Health Laboratories (APHL) staff, asking them to share their understanding of all aspects of the data and specimen flow with which they were familiar. We mapped major flows of respiratory specimens and test data contributing to virologic surveillance into a process map, tracing sources, routes, and destinations. We identified 5 virologic surveillance tiers in which specimens were collected or tested: outpatient care settings (tier 1), inpatient care settings and commercial laboratories (tier 2), state and local public health laboratories and health departments (tier 3), laboratories at CDC-sponsored National Influenza Reference Centers (NIRCs) (tier 4), and laboratories within the CDC National Center for Immunization and Respiratory Diseases Influenza Division (tier 5). We defined testing activities within each tier (Table) and incorporated them into a framework to characterize domestic influenza virologic surveillance. We used stakeholders’ reviews and comments for revisions to finalize the framework.

**Table:** Characteristics of influenza test types used for US domestic influenza virologic surveillance*

Characteristic	RIDTs†	Virus isolation	Direct fluorescent antibody tests	Molecular tests‡	Antiviral resistance functional tests	Antigenic tests§	Genetic sequencing
Result type	Influenza positive or negative AND type A or B (for most tests)	Virus growth	Influenza positive (type A or B), negative, or inconclusive	Influenza type and/or subtype positive, negative, or inconclusive	Resistant or not to adamantanes and neuraminidase inhibitors	Antigenic relatedness of viruses to vaccine or reference viruses	Genetic structure and relationship to previously circulating influenza viruses
Time to results	<30 min; most differentiate positive influenza A and B	Traditional: 3–10 d Rapid: 1–3 d	1–4 h	15 min–6 h	≈1 d	5–8 h	3–5 d (excluding isolation)
CLIA¶ category	Varies: CLIA-waived to moderate complexity	High complexity	Varies: moderate to high complexity	Varies: CLIA-waived to high complexity	High complexity	High complexity	High complexity

*CLIA, Clinical Laboratory Improvement Amendment; RIDT, rapid influenza diagnostic tests. †http://www.cdc.gov/flu/professionals/diagnosis/rapidlab.htm#table2. ‡http://www.cdc.gov/flu/pdf/professionals/diagnosis/molecular-assay-table-1.pdf. §Hemagglutination inhibition, microneutralization, and focus-reduction assays (https://www.cdc.gov/flu/professionals/laboratory/antigenic.htm). ¶ CLIA categories for laboratory complexity (https://wwwn.cdc.gov/clia/Resources/TestComplexities.aspx).

To observe trends in the type and amount of influenza testing performed in both outpatient and inpatient healthcare settings, we used MarketScan Research Databases (Truven Health Analytics, Atlanta, GA, USA) and Medicare and commercial carrier reimbursement claims, which provided test counts from 95,176,178 covered lives during 2010–2015 (*5*). We examined Current Procedural Terminology (CPT) codes for virus isolation (87252, 87253, and 87254), immunofluorescence (87275 and 87276), PCR (87501, 87502, 87503, 87631, 87632, 87633, and 87798), and rapid influenza diagnostic test (RIDT) (87400, 87449, 87804). We also used inpatient testing trends from published literature (*6*). To capture the volume and type of tests performed at clinical laboratories contributing to influenza virologic surveillance and at PHLs, we analyzed reports submitted to CDC from clinical providers, and state and local public health authorities participating in influenza surveillance. The CDC Influenza Division provided counts of surveillance tests performed in 3 CDC-supported NIRC laboratories and CDC laboratories.

## Results

**Figure 1:**
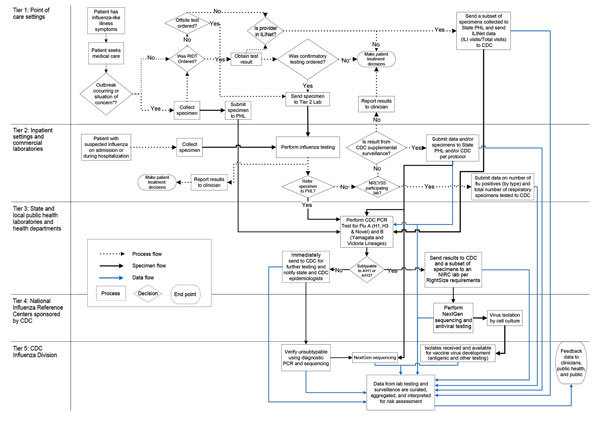
Influenza Virologic Surveillance Landscape illustrating the processes and the flow of specimens and test data through 5 tiers of testing activity. CDC, Centers for Disease Control and Prevention; ILI, influenza-like illness; ILINet, Influenza-Like Illness Surveillance Program; NGS, next-generation sequencing; NIRC, National Influenza Reference Center; NREVSS, National Respiratory and Enteric Virus Surveillance System; PHL, public health laboratory; RIDT, rapid influenza diagnostic test. *Situation of concern: epidemiologic factors indicating outbreak, potential for severe disease, resistant infection, or possible novel virus infection.

We categorized the multiple elements of the US domestic influenza virologic surveillance into the 5 tiers and captured the interrelationships of decisions and specimen and data submission in a process map (Figure 1). The tiers reflect the sequential flow of viruses, information, and location of activities contributing to virologic surveillance.

### Tiers 1 and 2

Tier 1 consists of outpatient care facilities, predominantly physician offices and urgent care centers. Specimens collected in this tier are used primarily for diagnosis and treatment decisions. Only a subset of care-seeking patients with influenza-like illness (ILI) will have respiratory specimens tested for influenza (*7*); of these respiratory specimens, only a subset is sent to the PHLs represented in tier 3. These specimens may be collected by ILINet providers and tier 1 providers designated by their state as influenza surveillance partners. ILINet is a network of >2,800 outpatient healthcare providers located in all 50 states, Puerto Rico, the District of Columbia, and the US Virgin Islands. Each week during influenza season, ≈2,000 ILINet provider-participants report total and ILI visits to CDC (*2*).

**Figure 2:**
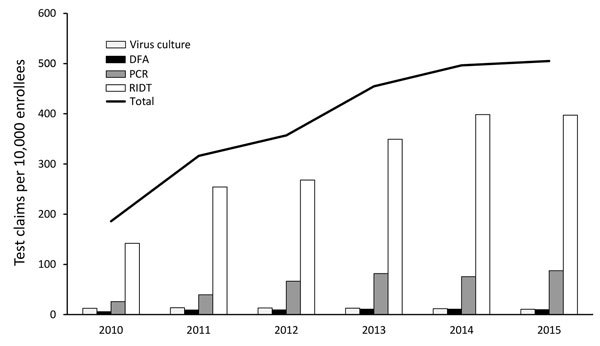
Number of influenza test claims per 10,000 enrollees in Truven Health Analytics’ Database 2010–2015, demonstrating that the total number of influenza tests has consistently increased, with RIDTs comprising the largest percentage of tests. DFA, direct fluorescent antibody test; RIDT, rapid influenza diagnostic test.

Clinicians may use many criteria when deciding whether and how to test for influenza. Physicians often test for influenza when there is a suspected outbreak in a facility or closed setting; when epidemiologic factors indicate the potential for severe disease; or when travel history, animal exposure, or both indicate possible infection with a potential pandemic virus. An analysis of claims data indicates that RIDTs were clinicians’ predominant testing choice during 2010–2015 (Figure 2) (*8*). Most RIDTs can be performed by healthcare providers in settings such as physicians’ offices or small clinics. Although RIDTs may exhibit high specificity, the suboptimal sensitivity of some RIDTs can produce false-negative results (*9*).

Tier 2 comprises laboratories with higher-complexity testing capabilities, such as hospital and commercial laboratories. Providers in tier 1 may send specimens to tier 2 laboratories to obtain more sensitive initial testing or as a follow-up to confirm RIDT results. These laboratories report test results to clinicians who can use them to validate or modify treatment decisions. However, tier 1 laboratories that participate in public health surveillance networks may also submit specimens directly to tier 3 for validation of results.

Tier 2 laboratories may forward a subset of specimens and data to PHLs in their jurisdictions as part of state-requested surveillance, or for further testing of unusual clinical cases, suspect novel events, or potential antiviral drug resistance. If the tier 2 laboratory is 1 of the ≈300 clinical laboratories that report test results through the National Respiratory and Enteric Virus Surveillance System (*2*), it will submit weekly counts of positive and total influenza tests to CDC.

Tiers 1 and 2 represent most influenza testing in the United States. The total number of influenza tests performed each year at tier 1 and 2 facilities is not known. Figure 2 shows the relative use and trend during 2010–2015 of 4 different influenza test types used in tiers 1 and 2. RIDT claims were ≈4 times more common than all other test claims combined, and the total number of test claims per 10,000 enrollees rose 2.5-fold during 2010–2015. In general, RIDTs are most frequently used in tier 1 facilities. Tests used in tier 2 facilities, such as virus isolation, direct immunofluorescence, and PCR, are generally more complex. PCR test claims increased >3-fold during 2010–2015. The estimated number of tests in the 2015–16 season from tier 2 that directly contributed to US influenza virologic surveillance was 804,000.

### Tier 3

Tier 3 comprises ≈100 state and local PHLs, in all 50 US states, that collaborate with CDC for influenza surveillance. These laboratories use standard CDC-supplied reverse transcription PCR (CDC RT-PCR) test reagents to detect influenza viruses A(H1N1)pdm09, A(H3N2), B/Yamagata lineage, B/Victoria lineage, A(H5N1), A(H7N9), and other novel influenza viruses. These laboratories test influenza specimens primarily for surveillance or outbreak investigations. Results are reported to state health departments and to CDC. A subset of specimens is also sent to CDC or a CDC-designated NIRC on the basis of guidelines established for each influenza season. Any specimen producing inconclusive results (i.e., influenza A with no subtype identified by CDC RT-PCR) may indicate infection with a novel influenza virus with epidemic or pandemic potential. These specimens are to be sent directly, as soon as possible, to CDC for rapid diagnostic confirmation and comprehensive characterization with notification to state and CDC epidemiologists. Some PHLs participate in 1 of 3 supplemental surveillance systems that ask participants to send additional influenza-positive specimens, related data, or both to CDC. Supplemental surveillance systems include CDC’s Influenza Hospital Surveillance Network (FluSurv-Net), the Influenza Incidence Surveillance Program, and the US Influenza Vaccine Effectiveness Network. FluSurv-Net monitors hospitalizations related to laboratory-confirmed influenza in 13 states (>70 counties) (*1*). The Influenza Incidence Surveillance Program consists of a convenience sample of primary outpatient practices in 6 states, recruited by their state health departments, that collect respiratory specimens from all ILI patients (*10*). The US Influenza Vaccine Effectiveness Network includes ambulatory care facilities affiliated with 5 major medical centers in Washington, Wisconsin, Michigan, Pennsylvania, and Texas. These facilities provide data and specimens to CDC from patients seeking care for acute respiratory infections (*11*).

**Figure 3:**
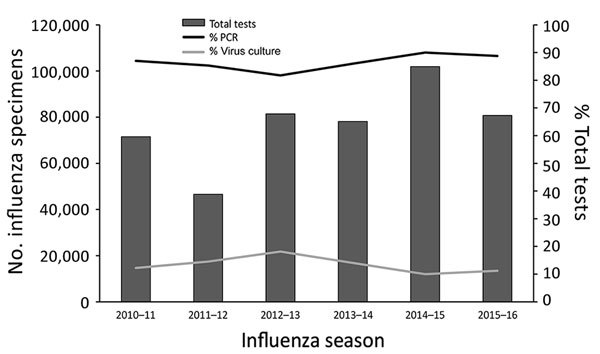
Number of influenza tests reported by public health laboratories to CDC since 2010. The number of specimens tested varies with the severity of the season. Since 2010, an average of 77,000 specimens has been tested annually. Multiple tests may be performed on a single specimen. Most tests have been PCR.

Tier 3 laboratories tested ≈78,000 respiratory specimens during the 2015–16 influenza season. During 2009–2016, the number of tests reported to CDC by public health laboratories varied by season; most reported tests were PCR, using the CDC RT-PCR assay (Figure 3). Virus culture was performed less frequently. Tier 3 laboratories are key for novel influenza A virus detection. In addition to testing for all currently circulating human influenza viruses, the CDC RT-PCR allows tier 3 laboratories to presumptively identify human infection with swine variant influenza viruses and avian influenza A(H5N1) and A(H7N9) viruses. CDC training of the tier 3 laboratories and the use of common platforms and test methods permit rapid deployment of new assays in the event of a public health emergency.

### Tier 4

Tier 4 consists of 3 state PHLs designated by CDC as NIRC laboratories. These laboratories receive specimens from tier 3 PHLs, isolate viruses in cell culture to sufficient volumes and titers, and assess susceptibility of viruses to antiinfluenza medications. The number and influenza type/subtype of specimens sent from tier 3 PHLs is determined using an online calculator tool for determining each jurisdiction’s sample size for submission. This effort, termed rightsizing, began implementation in 2013 and was used for the 2015–16 season in all submitting jurisdictions in all 50 US states (*12*).

**Figure 4:**
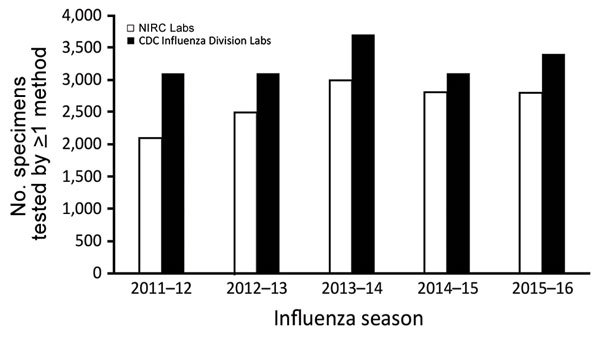
Number of influenza specimens tested for domestic surveillance in tier 4 (NIRCs) and tier 5 (CDC, Atlanta) laboratories. NIRCs receive specimens from tier 3 laboratories and are a major source of specimens for tier 5 laboratories. CDC, Centers for Disease Control and Prevention; NIRC, National Influenza Reference Center.

Since 2015, NIRC laboratories have also begun using next-generation whole-genome sequencing (NGS) directly from clinical specimens to characterize viruses. Sequence data from NIRC laboratories are immediately available to CDC during sequence runs through a cloud-based sequence analysis platform. All remaining original clinical specimens and virus isolates are sent to CDC for further characterization. Since 2010, from 2,100 to 3,000 specimens from domestic surveillance have been tested in tier 4 each season (Figure 4). For the 2015–16 season, 2,800 specimens were tested in tier 4.

### Tier 5

Tier 5 represents laboratories at CDC. These laboratories receive specimens and isolates from the NIRCs and specimens collected by PHLs during case or outbreak investigations from clinicians concerned about novel or drug-resistant influenza virus infections in humans. Laboratories at CDC also receive specimens and isolates from international laboratories for virologic surveillance. Viruses that have not undergone NGS at a NIRC undergo NGS in CDC laboratories. CDC scientists analyze all genetic sequencing data to identify viruses of epidemiologic and clinical importance. Final gene sequences and related information are submitted to the GISAID database (*13*) and GenBank (*14*). GenBank produces an annotated collection of all publicly available DNA sequences. The GISAID initiative focuses exclusively on influenza viruses and provides open access to sequences, clinical and epidemiologic data, and geographic data.

Influenza viruses from the NIRCs and those sent directly to CDC are characterized by hemagglutination inhibition, microneutralization, or focus-reduction assays to determine antigenic relatedness to vaccine viruses. Specimens sent directly to CDC are also tested for susceptibility to antiviral drugs. Supplemental PCR testing is done on specimens for which subtyping performed elsewhere is inconclusive or requires confirmation. CDC scientists analyze all results to identify viruses of epidemiologic and clinical importance and to prepare information documents for the WHO twice-yearly consultation meetings on composition of influenza vaccines.

CDC epidemiologists and laboratorians aggregate and analyze data from PHLs, NIRCs, and other designated surveillance laboratories; outpatient illness data; influenza-associated hospitalization and mortality data; and state epidemiologist reports of the geographic spread of influenza to identify currently circulating viruses and their clinical impact. These data are used to produce FluView (*15*), a weekly surveillance report, as well as other communication products that share surveillance data with clinicians, public health officials, and the public. The data are also used for periodic risk assessments of newly emerging novel influenza viruses (*16*).

Multiple influenza tests are conducted in tier 5. During the 2015–16 season, CDC’s influenza laboratories tested ≈3,400 influenza viruses from the domestic surveillance system; most of these viruses were received from tier 4 NIRCs for additional analysis (Figure 4). Specimens collected during case and outbreak investigations were also submitted to CDC, as were specimens submitted by tier 3 laboratories when results generated using the CDC RT-PCR required confirmation or advanced laboratory testing. Nearly 70% of viruses received were antigenically characterized by hemagglutination inhibition. A subset, including those unable to be tested with hemagglutination inhibition, was subsequently tested in supplementary microneutralization assays. Almost all specimens received directly underwent NGS. The percentage of specimens with a record of sequencing activity increased from 27% in 2011 to 100% in 2016. Sequencing before 2015 included some traditional Sanger sequencing of the hemagglutinin (HA) and neuraminidase (NA) (and sometimes matrix [M]) gene segments.

Combining information from all tiers during the 2015–16 influenza season, CDC reported ≈804,000 influenza test results from tier 2 National Respiratory and Enteric Virus Surveillance System laboratories, 78,000 from tier 3 PHLs, 2,800 from tier 4 NIRCs, and 3,400 from CDC laboratories.

## Discussion

The US influenza virologic surveillance landscape is a system that has developed over >40 years. In 1973, US virologic surveillance consisted of 60 cooperating laboratories mailing weekly reports to CDC of specimens submitted and specimens positive for influenza isolation (*17*). The current system includes a much wider compilation of participants and laboratory practices, including NGS and automated electronic laboratory reporting. Using several data sources, we developed a framework and process diagram of 5 testing tiers to assist efforts of diagnostic modelers, public health officials improving the efficiency of surveillance, and agencies revising pandemic plans.

The US influenza virologic surveillance landscape is complex. A diversity of system participants, each functioning with its distinct testing purposes, sampling approaches, testing algorithms, and test methods, contributes to complexity both within and between the tiers (Figure 1). The purpose of influenza testing influences the amount of testing performed and the test methods. Ultimately, all influenza virologic surveillance relies on specimens collected from symptomatic patients during medical encounters in tiers 1 and 2, where the purpose of testing is primarily patient diagnosis rather than surveillance. Influenza tests are most useful for individual patients when likely to give results helpful for diagnosis and treatment decisions. The decision to test ILI outpatients for influenza is based on the individual physician’s knowledge, background, experience, and interest, as well as current influenza activity, resulting in diverse testing practices. Specimens are collected and subsequently available for public health surveillance from only a subset of patients seeking medical care, which in turn is a subset of those experiencing symptoms. Data obtained from test results in tier 1, therefore, may be subject to bias. As tests improve in sensitivity and specificity, samples from more patients may be tested and found positive, potentially leading to better treatment and illness outcomes, as well as improvements in the quality of influenza surveillance.

The types of tests in the diagnostic landscape are changing. Because RIDTs are the least expensive influenza tests, do not require complex testing capabilities, and can be performed in most physicians’ offices and outpatient clinics, they continue to comprise the greatest percentage of tier 1 influenza tests. However, the use of molecular (e.g., PCR) tests has increased. According to a survey of 931 clinical laboratories, the adoption of molecular test methods, some of which subtype influenza viruses, detect multiple respiratory pathogens, or both, more than doubled during the 2009 H1N1 pandemic (*18*). Increases in the use of PCR in tier 2 were also observed in FluSurv-NET, rising from <10% during 2003–2008 to ≈70% during 2009–2013 (*6*). Our data show that overall influenza testing increased 2.5-fold and PCR testing increased >3-fold during 2010–2015 (Figure 2). These increases may be the result of an increase in awareness of influenza following the 2009 pandemic and may be attributable to physician demand for more sensitive and specific influenza diagnostics.

One notable change in testing is evident in tiers 4 and 5, where NGS is now routine. In 2015, CDC began the Sequence First Initiative to introduce NGS for all specimens sent to CDC for virologic surveillance. The project continued in 2016, as NGS began to be implemented at CDC-supported NIRCs. As of August 2017, all specimens tested at CDC or NIRCs undergo NGS using bioinformatics and computational science, both at CDC and through cloud services, for rapid data sharing and analysis. NGS reveals the genetic variation among different virus particles in a single specimen and allows public health laboratorians to confirm the genetic identity of circulating viruses (*2*). These sequence data are also now a critical component of the twice-yearly WHO influenza vaccine virus selection process and are used in molecular modeling and forecasting. As the cost of NGS drops and the availability of more rapid sequencing platforms increases, NGS may begin to serve as a routine approach for influenza virologic surveillance in tier 3 laboratories as well (*19*).

Specimen collection and testing practices were found to vary across tier 3 state and local PHLs; however, new efforts have introduced a more standard approach for surveillance at the tier 4 and 5 levels. Since 2013, PHLs have been able to access right-size calculators to calculate an optimal number of specimens required for effective surveillance. These calculators, developed through a collaborative effort between CDC and APHL, use statistical tools to determine the amount of testing required for desired confidence levels of surveillance (*12*). These calculators allow state and local PHLs to evaluate their virologic surveillance systems and to improve the efficiency, representativeness, and timing of specimen submissions to CDC (*20*). Through rightsizing the submission of specimens, CDC now has a more systematic approach to identifying early drift in seasonal influenza viruses, detecting unsubtypable and potentially pandemic viruses, and selecting more representative and timely viruses for use in annual influenza vaccines.

Finally, we have provided an operating framework of specimen testing and surveillance that can support pandemic planning and response efforts. In 2017, the US Department of Health and Human Services released an update to the Pandemic Influenza Plan originally released in 2005, prompting the need for revised operational plans at the federal, state, and local levels (*4*). A critical component of those plans will require outlining how early detection and reporting of influenza viruses will be executed to respond rapidly to an emerging pandemic. The virologic surveillance landscape provided here delineates the various public health agency roles and responsibilities for virologic surveillance for seasonal, as well as pandemic, influenza. In addition, the landscape framework, along with the described rightsize calculators, provides estimates for specimens tested at each tier and ways to determine how many specimens are expected and needed during surge. CDC will also use the framework to estimate the needed number of PCR reagent kits it distributes from the International Reagent Resource (*21*) to the nearly 100 PHLs in the United States that participate in tier 3 virologic surveillance. Finally, resource, reimbursement, and logistics modelers can use the framework and estimates for developing or revising tools for use by planners and response agencies.
